# Fluorescent Light Incites a Conserved Immune and Inflammatory Genetic Response within Vertebrate Organs (*Danio rerio*, *Oryzias latipes* and *Mus musculus*)

**DOI:** 10.3390/genes10040271

**Published:** 2019-04-03

**Authors:** Mikki Boswell, Yuan Lu, William Boswell, Markita Savage, Kim Hildreth, Raquel Salinas, Christi A. Walter, Ronald B. Walter

**Affiliations:** 1 The *Xiphophorus* Genetic Stock Center, Department of Chemistry and Biochemistry, Texas State University, San Marcos, TX 78666, USA; mb77@txstate.edu (M.B.); y_l54@txstate.edu (Y.L.); wb1016@txstate.edu (W.B.); markita@txstate.edu (M.S.); raquel.salinas@txstate.edu (R.S.); 2 Department of Cellular Systems and Anatomy, The University of Texas Health San Antonio, San Antonio, TX 78229, USA; hildreth@uthscsa.edu (K.H.); walter@uthscsa.edu (C.A.W.)

**Keywords:** gene expression, RNA-Seq, fluorescent light, acute phase, oxidative stress

## Abstract

Fluorescent light (FL) has been utilized for ≈60 years and has become a common artificial light source under which animals, including humans, spend increasing amounts of time. Although the solar spectrum is quite dissimilar in both wavelengths and intensities, the genetic consequences of FL exposure have not been investigated. Herein, we present comparative RNA-Seq results that establish expression patterns within skin, brain, and liver for *Danio rerio*, *Oryzias latipes*, and the hairless mouse (*Mus musculus*) after exposure to FL. These animals represent diurnal and nocturnal lifestyles, and ≈450 million years of evolutionary divergence. In all three organisms, FL induced transcriptional changes of the acute phase response signaling pathway and modulated inflammation and innate immune responses. Our pathway and gene clustering analyses suggest cellular perception of oxidative stress is promoting induction of primary up-stream regulators *IL1B* and *TNF*. The skin and brain of the three animals as well as the liver of both fish models all exhibit increased inflammation and immune responses; however, the mouse liver suppressed the same pathways. Overall, the conserved nature of the genetic responses observed after FL exposure, among fishes and a mammal, suggest the presence of light responsive genetic circuitry deeply embedded in the vertebrate genome.

## 1. Introduction

Over three billion years of evolution occurred exclusively under sunlight. Thus, all spectral wavelengths were represented, and each organism had the opportunity to adaptively pair genetic responses with discrete regions and/or intensities of solar spectrum wavelengths inherent to their environmental niche. In contrast to the solar spectrum, fluorescent light (FL) has only been used for ≈60 years. The FL light spectrum is comprised of a much narrower range of wavelengths and exhibits sharp peaks and valleys of intensity for select wavelengths, when compared to the broad and consistent solar spectrum. We recently characterized changes in gene expression after FL exposure to be both robust and conserved in the skin of three divergent small fish species; *Xiphophorus maculatus* (platyfish), *Danio rerio* (zebrafish), and *Oryzias latipes* (medaka), that are utilized as experimental models in biomedical research [1,2]. In addition, we have reported that exposure to select wavelengths of light may serve to induce, or suppress, specific genetic pathways in *Xiphophorus* skin [3]. These studies established that exposure to one wavelength may activate genes in specific biochemical pathways, while exposure to a different wavelength of light can be used to suppress the same pathway. These studies support the concept that, over evolutionary time, each wavelength in the solar spectrum may have been conscripted for regulation of specific genetic pathways. If so, exposure to the full complement of solar wavelengths may be necessary to produce the agonistic/antagonistic genetic signaling needed for proper interaction of an organism with its environment.

Use of broad-spectrum artificial lighting has been shown to have the potential to produce adverse effects on human health, including depression, disturbed sleep–wake cycles, obesity, altered body temperature, and altered circadian rhythms [4,5,6,7,8,9,10,11]. However, little research has been conducted to determine genetic consequences, if any, from use of common artificial light sources. We have reported that exposure to FL in the skin of both zebrafish and medaka modulates gene expression patterns consistent with activation of inflammation and cellular immune responses [1]. However, in addition to the conserved primary response, each fish also shows other light-induced gene expression alterations that are unique to each species. Such species-specific genetic responses to the same FL source may reflect genetic fine-tuning of the organismal light response, according to evolution within unique environmental niches. This may suggest ocular photoreception and the genetic perception response to light are overlapping but separate processes.

Additional evidence for differences in the genetic perception of light have also been observed in male and female *X. maculatus* exposed to ultraviolet light (UVB, 311 nm, [12]). When highly inbred siblings (i.e., over 100 generations) are exposed to the same UVB light source, most of the induced gene expression responses are shared by both sexes. However, males and females also show opposite UVB induced modulation of gene sets in important pathways, such as synaptic development, cell differentiation, wound healing, glucose metabolism, and free radical scavenging. Thus, gender differences within a species may affect the genetic response to light exposure. While these studies document some differences in the specific gene sets modulated after light exposure, it is important to note that for skin, the primary consequence of FL exposure for all fish species examined appears to a be well-conserved induction of the inflammation and immune response.

Previous studies of the genetic response to FL, UVB, or specific wavelengths of light have involved only the skin of several fish models. Potential transcriptional effects within internal organs after light exposure of intact animals has not been reported. In addition, fish skin is a genetically dynamic organ compared to the skin of mammals. Thus, it is of interest to determine whether the conserved fish gene expression responses to FL exposure would extend to the skin of mammals.

Herein we report results of comparative studies using RNA-Seq to assess modulation of transcriptional profiles following exposure to 4100 K “cool-white” FL for two commonly utilized fish models (zebrafish and medaka) and a mammalian model (the hairless mouse). FL induced responses in gene expression were measured in skin, a direct light receiving organ, and two internal organs, the brain and liver. In spite of considerable evolutionary divergence (i.e., ≈450 My) and drastically different lifestyles (i.e., diurnal fish and nocturnal mice), a highly conserved primary genetic response to FL exposure was observed. In skin, brain, and liver of all three animals, FL exposure led to modulation of the acute phase response pathway, with concurrent promotion of inflammation and immune responses. The only major difference in the primary response to FL was observed in the mouse liver, that modulated the same pathways as the fish livers, but in the opposite direction compared to the fishes (i.e., suppression in mouse liver, induction in fish livers). These findings support the concept that light-induced genetic circuitry is highly conserved and deeply embedded in the vertebrate genome.

## 2. Materials and Methods

### 2.1. Fish Utilized and Fluorescent Light Exposure

Mature adult male *Danio rerio* (TU, zebrafish, 3) from the Zebrafish International Resource Center in Eugene, OR, and three mature adult male *Oryzias latipes* (Cab, medaka) from the *Xiphophorus* Genetic Stock Center were used for FL exposure (40 min, 35 kJ/m^2^). All protocols were carried out as previously described [1,13]. Prior to light exposure, fish were placed individually into 100 mL of filtered home aquaria water and kept in the dark for 14 h. FL exposure occurred in UV-transparent cuvettes (9 × 7.5 × 1.5 cm) in 90 mL of water. The exposure cuvettes were suspended 10 cm between two banks of two (total of 4 lights) unfiltered 4,100 K fluorescent lights (Philips F 20T12/CW 20 watts, Alto, 40 min, 35 kJ/m^2^), mounted horizontally on each side of a wooden box exposure chamber. After FL exposure, all fish were returned to the dark in 100 mL filtered aquaria water for 6 h and then euthanized and dissected for RNA isolation. All organs were dissected into 300 μL RNA*later* (Life Technologies, Grand Island, NY, USA) and stored in the −80 °C freezer except for the skin samples, which were immediately placed in 300 μL TRI Reagent (Sigma Inc., St Louis, MO, USA) and flash frozen in an ethanol dry ice bath. In addition, 3 fish of each species were placed into the cuvettes following the procedure outlined above and placed into the exposure chamber but with the lights turned off (sham treated fish). All other protocols described above were followed for the sham-treated samples.

### 2.2. Mice Utilized and Fluorescent Light Exposure

Three young (7 weeks old) adult male hairless mice (*M. musculus*, SKH1-ELITE, Charles River Laboratories, Wilmington, MA, USA) were used for FL exposure (60 min, 52.5 kJ/m^2^). The mice were housed and cared for at the University of Texas Health San Antonio; all experimental treatments and protocols were performed to mimic the fish exposures but adjusted for the larger body mass. Prior to FL exposure, all mice were housed in cages, each containing 2 mice per cage, and placed into a dark cabinet in the exposure room. Mice were placed, individually, into round quartz glass chambers, 8 cm in diameter with 6-cm high sidewalls. These chambers allowed the mice to turn around and/or move freely. During light exposure, each chamber, with a single mouse, was covered with mesh screening and placed into the same light box utilized for fish exposures (see above), so the center of the chamber wall was the same distance from the bottom of the exposure box bottom as the fish exposure cuvettes. Following FL treatment, the mice were placed back in their cages and returned to the dark for 4 h. Three sham-treated mice were subjected to the same protocol outlined above, but the FL lights were off. All mice were sacrificed following the 4 h time period using cervical dislocation and dissected into organs. All organs were placed into pre-labeled 15 or 50 mL conical vials containing RNA*later*, corresponding to the size of the organ. Vials were placed on dry ice until after dissections were complete and stored in the −80 °C freezer until RNA was subsequently isolated.

### 2.3. Statement of Animal Use

All medaka and zebrafish utilized in these studies were maintained in the *Xiphophorus* Genetic Stock Center (http://www.xiphophorus.txstate.edu/) in accordance with protocols approved by the Institutional Animal Care and Use Committee (IACUC, IACUC#2016103230 and IACUC#20173294956). These fish were maintained in accordance with the applicable OLAW guidelines governing animal experimentation in the USA. Mice were maintained and treated in an AAALAC-accredited animal facility at The University of Texas Health San Antonio, according to IACUC approved protocols.

### 2.4. RNA Isolation and Sequencing

Total RNA was isolated from skin, brain, and liver of zebrafish, medaka, and mouse samples using a TRI Reagent (Sigma Inc., St Louis, MO, USA) chloroform extraction followed by the Qiagen RNeasy (Qiagen, Valencia, CA, USA) isolation protocol. Skin was homogenized in 600 μL TRI Reagent using a handheld tissue disruptor, followed by addition of 120 µL of chloroform. Samples were vigorously shaken and then phases partitioned by centrifugation (12,000× *g* for 15 min at 4 °C). After extraction, the RNA was precipitated with 500 μL 70% EtOH and further purified using a Qiagen RNeasy mini RNA kit following the manufacturer’s protocol. Residual DNA was eliminated with an on-column DNase treatment at 25 °C for 15 min. RNA quality was assessed with an Agilent 2100 bioanalyzer (Agilent Technologies, Santa Clara, CA, USA), and quantified with a Qubit 2.0 fluorometer (Life Technologies, Grand Island, NY, USA). All samples sent for sequencing had RIN scores ≥8.0.

### 2.5. Differentially Expressed Gene (DEG) Analysis

RNA sequencing was performed on libraries constructed using the Illumina TrueSeq library preparation system that employs a polyA selection. RNA libraries were sequenced as 100 bp paired-end fragments using an Illumina Hi-Seq 2000 system (Illumina, Inc., San Diego, CA, USA). Forty-seven to 90 million raw reads were generated for each RNA sample. All raw reads were subsequently truncated by similarity to remove library adaptor sequences using a custom Perl script, and short reads were filtered based on quality scores using a custom filtration algorithm that removed low-scoring sections of each read and preserved the longest remaining fragment (Table S2) [14]. Filtered reads were mapped using Tophat2 [15] to the corresponding *Danio rerio* (zv9), *Oryzias latipes* (medaka1.81.2), or *Mus musculus* (mm10) genome. The percentage of reads mapped were assessed by Tophat2, and sequencing depth assessed by SAMtools depth, respectively [15,16]. Gene expression was assessed by featureCounts using genome annotation from Ensembl database v79 [17], and differentially modulated genes were determined using the R-Bioconductor (www.bioconductor.org) package edgeR [18] with a |log_2_(fold change)| ≥ 1.0 (FDR < 0.05), (Table 1).

Genes identified as being differentially modulated in response to FL were further analyzed for organ specificity using Venny 2.1 [19] and for functional specificity with Ingenuity Pathway Analysis (IPA, Qiagen, Redwood City, CA, USA). IPA-based gene expression analysis yielded gene clusters, genetic pathways, functional classes, and potential up-stream regulators to aid in mechanistic interpretation. Herein, the term “pathways” is short for canonical pathways assigned by IPA based on the light exposure input differentially expressed gene (DEG) data. In IPA, known pathways are drawn as pictures with input DEGs overlaid onto them that are identified by symbols and colors indicating known functions and direction of modulation. A z-score algorithm is used to determine if a pathway is up- or down-regulated based on the genes that fall into that particular pathway and the direction of modulation. IPA assignment of DEGs into “functions” or “functional classes” relates the input DEGs to known disease states and biological functions, as published in the scientific literature. Functional classes are visualizations of the biological trends in the light-effected DEG dataset, and may be used to predict the effect of gene expression changes of the entire dataset on biological processes and known cellular functions. Functional assignment uses an algorithm to assess the dataset as a whole and predict what is collectively occurring on a larger down-stream scale.

### 2.6. Validation of RNA-Seq Gene Expression Results

NanoString (NanoString Technologies, Inc., Seattle, WA, USA), with a custom panel for zebrafish (Table S1) and a separate panel for medaka [20], was used as an independent technology to confirm the DEGs identified using RNA-Seq. For mouse, a commercially available nCounter PanCancer Immune Profiling Panel (NanoString, https://www.nanostring.com, XT-CSO-MIP1-12) was used for RNA-Seq validation. Aliquots of the RNA (500 ng) used for RNA-Seq were also used for the NanoString nCounter assay. Hybridization protocols were strictly followed according to manufacturer’s instructions [21]. Samples were hybridized overnight at 65 °C with custom probes and transferred to the NanoString Prep Station. The NanoString cartridge containing the hybridized samples was immediately evaluated with the NanoString nCounter based on unique color-coded signals. Probe counts were quantified through direct counting with the nCounter Digital Analyzer. Data analysis was performed by lane normalization using a set of standard NanoString probes, followed by sample normalization using a set of 10 housekeeping genes. Fold changes were calculated on normalized counts and plotted using Microsoft Excel.

All RNA-Seq short read sequence data utilized to prepare the differential expression analyses presented herein are deposited on the *Xiphophorus* Genetic Stock Center website (https://www.xiphophorus.txstate.edu) and will be made available upon reasonable request to the corresponding author. The final differential expression gene lists are published with the supplementary data files associated with this manuscript.

## 3. Results

**Figure 1:**
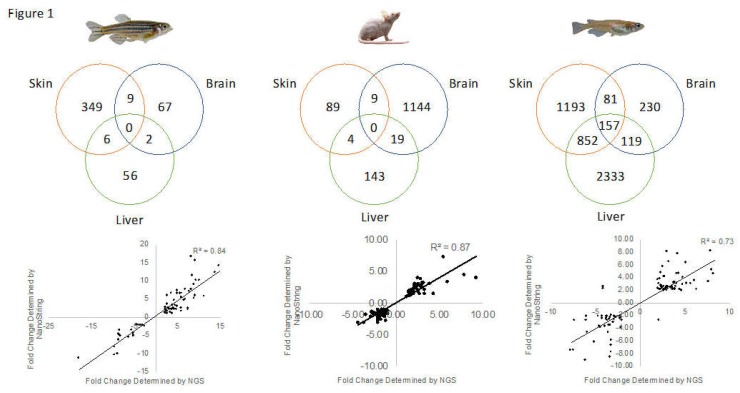
Following FL exposure, modulated genes compared to sham-treated samples were converted into HUGO IDs and imported into IPA. The mapped IPA genes from each organ tested (skin, brain, and liver) were compared using Venny (top row). Gene expression was verified using NanoString nCounter assay, and comparing fold changes back to RNA-Seq determined fold changes for each model tested (bottom row). In zebrafish (left) a total of 72 targets were tested using the nCounter assay on a custom zebrafish panel (Table S1). A Mouse PanCancer Immune Profiling Panel was used, and 103 gene targets were above background (center). In medaka (right), a total of 373 targets were tested using the nCounter assay on a custom medaka panel.

Adult male zebrafish, medaka, or hairless mice were exposed to FL (35 kJ/m^2^ or 52.5 kJ/m^2^) as previously detailed [1,13,22,23]. Isolated total RNA from all organ samples was subjected to Illumina sequencing, as previously described [1,3,12,13,22,23,24]. Table S2 shows RNA-sequencing (RNA-Seq) statistics for the reads utilized in this report. As shown, the RNA-Seq read lengths were quite deep, ranging from 3.42 × 10^9^ (medaka sham, liver) to 12.9 × 10^9^ (mouse FL, liver), post filtration. This equates to exome sequencing depths ranging from 147× to 215×, with most samples well over 120× (Table S2). The numbers of DEGs for each of the organs after FL exposure, for each species examined, is shown in Table 1. Table S3 presents a list of all DEGs for skin in zebrafish (S3a), medaka (S3b), and mice (S3c), while supplementary Tables S4a–c and S5a–c, present DEG lists for brain and liver, respectively. Medaka skin (2304 DEGs) and liver (3493 DEGs) showed the most robust genetic responses to FL, compared to zebrafish (23 and 83, respectively) or mice (107 and 182, respectively). However, the mouse brain exhibited the highest numbers of brain DEGs (1,174) compared to medaka (592) and zebrafish (94). For all three organisms, there was very little overlap in DEGs, when gene I.D.s for each organ from one of the test animals was compared to the other two (Figure 1, top). Medaka showed the highest degree of overlap after FL exposure with 157 DEGs shared by all three organs (skin, brain and liver), while in zebrafish and mice showed no overlap among all three organ targets (Figure 1).

DEGs identified by RNA-Seq after FL exposure were validated by independent measurement of gene expression using the NanoString n-counter platform [21] as previously detailed [1,3,12,13,22,23,24,25]. Using the NanoString assay, 72 zebrafish targets (30 targets in skin, 18 in brain, and 21 in liver) were tested and 69 were above background. All (100%) of these matched the RNA-Seq DEGs in direction of modulation with an R^2^ = 0.84 (Figure 1, left bottom). For medaka, 373 targets were tested using the nCounter assay (117 in skin, 41 in brain, and 66 in liver) and 100% matched the RNA-Seq in direction with an R^2^ = 0.87 (Figure 1, right bottom).

Validation of RNA-Seq results in mice skin, brain, and liver employed the commercial NanoString Mouse PanCancer Immune Profiling Panel that simultaneously tested the gene expression profile for 800 gene targets including 50 housekeeping genes [26]. In total, 103 gene targets on the PanCancer panel were found informative and statistically valid in the mouse RNA-Seq dataset, with 97% of these targets confirmed in modulation direction (all but three) and an R^2^ = 0.73 (Figure 1, middle bottom). Two mouse liver targets, and one brain target, did not agree in direction among the 29 skin, 39 brain, and 35 liver target genes tested.

### 3.1. Genetic Response of Skin to Fluorescent Light

Zebrafish skin induced 364 genes (Table S3a), which Ingenuity Pathway Analysis (IPA, Qiagen, Redwood City, CA, USA) clustered into 19 canonical pathways having |z-scores| > 2.0 (12 up-modulated and seven down-modulated, Table 2, top). In comparison, adult male medaka exposed to FL modulated 2284 genes (Table S3b) in skin associated with 24 canonical pathways (19 up-modulated and five down-modulated, Table 2, middle), while mature male hairless mouse skin, exhibited 102 DEGs (Table S3c) that clustered into 12 up-modulated canonical pathways (Table 2, bottom).

**Figure 2:**
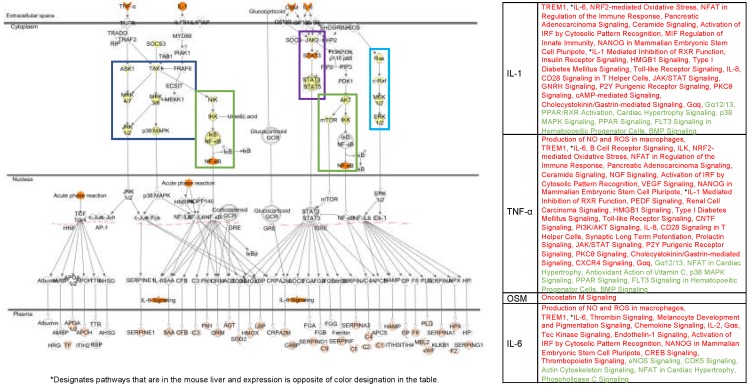
The acute phase response (APR) is central to the FL induced response in skin, brain, and liver of all three model organisms. Four main regulators (*IL1B*, *TNF*, *OSM*, and *IL-6*) are central to this response (top of pathway in dark orange). The expression of these regulators controls four major signaling cascades, the MEK/MKK cascade (dark blue), the *NFkβ* cascade (green), the JAK/STAT cascade (purple), and the Ras/Raf cascade (light blue). The pathways from Table 2, Table 3 and Table 4 that overlap with the APR (designated AP in each table) all fall into one of these signaling cascades under the control of one of the four primary regulators (Table on the right). The products following the APR are highlighted in light orange and each helps induce an inflammatory and/or immune response in the organism. Pathways in the table on the right are in red font if they are up-modulated and green font if they are down-modulated. Pathways labeled by an * are in the mouse liver as well as other organs tested. The color indicated for these pathways follows the primary response and is; therefore, opposite in the mouse liver.

The top canonical pathway in zebrafish skin (based on z-score and pathway coverage) was the acute phase response (APR, Table 2, Figure 2, [27,28]). Overall, based on the DEG clustering following FL exposure, zebrafish skin responded by mounting a robust inflammation and immune response that principally involved modulation of sub-pathways within the APR (Table 2, Figure 2, [27,28,29,30]). The APR is a large stress pathway that includes many sub-pathways, such as Oncostatin M, mTOR, PPARα/RXRα, TREM1, and others (Figure 2). All of the APR sub-pathways showed significant z-scores in zebrafish skin (Table 2, Figure 2), and all but three of the 21 pathways modulated by FL were associated with inflammation and the APR (Table 2).

Of the 24 significantly modulated pathways identified in medaka skin after FL, 18 were associated with inflammation, immune, or the APR (Table 2, middle). Seven of the FL significantly modulated canonical pathways in medaka skin (i.e., PPARα/RXRα activation, B cell receptor signaling, TREM1 signaling, ILK signaling, IL-6 signaling, Gα12/13 signaling, and the APR) were shared with zebrafish skin and were modulated in the same direction, with the exception of Gα12/13 signaling.

For the hairless mouse FL induced DEGs, 11 of the 12 pathways modulated in mouse skin were also observed to be involved in inflammation, immune, or the APR responses (Table 2, bottom). Pathways shared with the zebrafish skin FL response included APR, PPARα/RXRα activation, leukocyte extravasation signaling, and TREM1.

**Figure 3:**
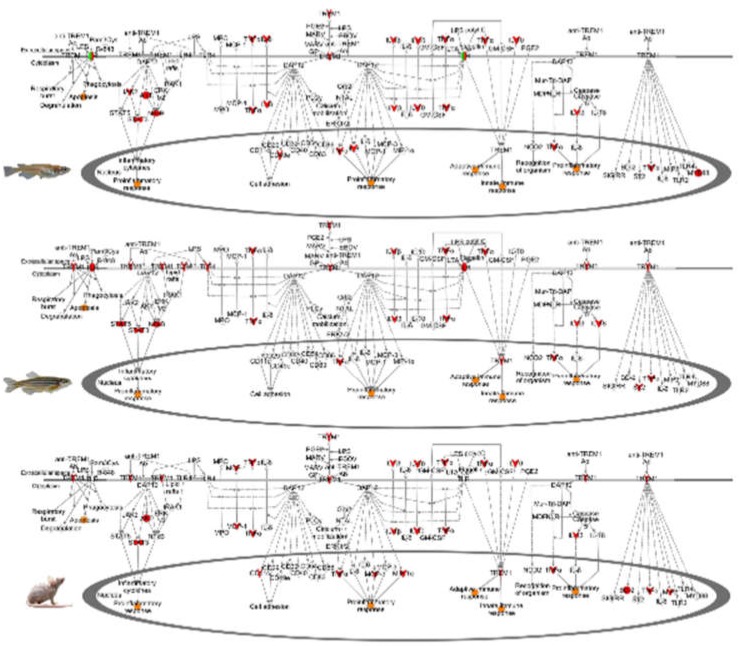
TREM1 modulation in medaka (top), zebrafish (middle), and mouse (bottom) show considerable overlap following FL exposure. All three vertebrates up-modulate key regulators, including *TNF* and *IL1B*. Red genes are up-modulated and green genes are down-modulated. Predicted down-stream functional effects that are expected to increase are in orange and to decrease are in blue.

Zebrafish medaka and mouse skin exposed to FL shared three canonical pathways (TREM1 signaling, APR signaling, and PPARa/RXRa activation). Of these, the most significantly up-modulated pathway following FL exposure in mouse skin was the TREM1 signaling pathway, a sub-pathway of the APR. For the TREM1 pathway, both zebrafish and mouse showed 12 genes modulated (41% coverage), with z-scores of 3.0 and 4.2, respectively, while medaka modulated 13 genes with a z-score of 2.4 (Table 2, Figure 3). As an example of the conserved FL response in skin, the TREM1 pathway for the two fish, and the mouse, is compared in Figure 3. Here we observed many of the same gene targets modulated similarly in all three organisms after FL exposure. The TREM1 pathway, part of the APR (Figure 2), lead to the initiation of the JAK/STAT pathway, ERK/MAPK signaling, as well as the production of many of the cytokines that enhance the immune and inflammation responses (*IL-8*, *TNF*, *IL1B*, *IL-6*).

**Figure 4:**
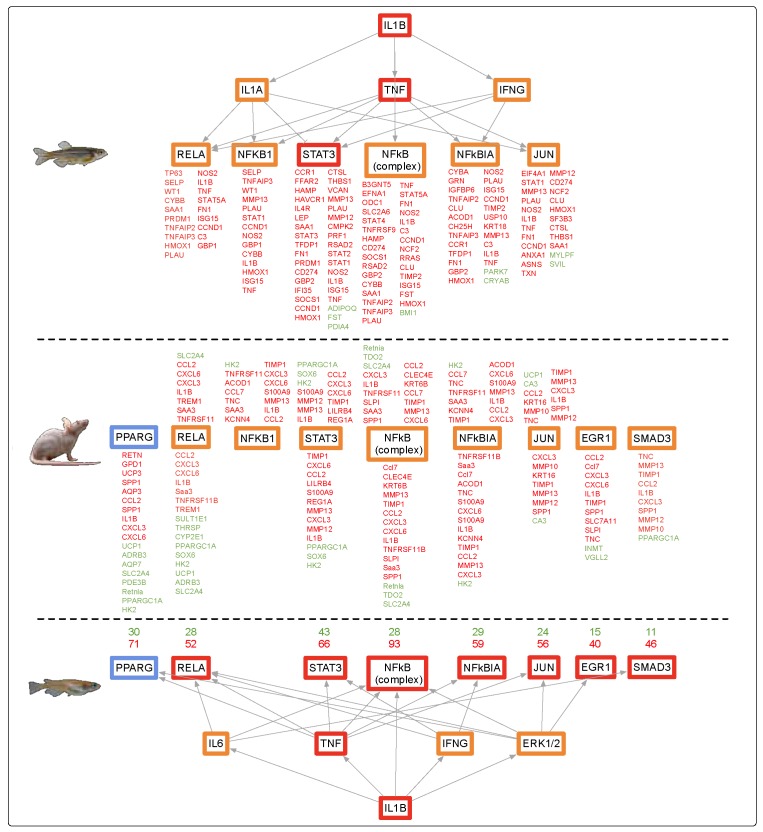
*IL1B* controls expression of many shared mid-level regulators in the zebrafish (top), medaka (bottom), and mouse (middle) skin datasets, including the key immune and inflammation APR regulator *TNF*. Genes in red and green are modulated differentially expressed genes (DEGs) and genes in orange and blue are predicted by IPA to be up and down, respectively. Due to the robust dataset in medaka, the number of genes up- and down-modulated are listed in red and green, respectively.

The skin of zebrafish, medaka, and mice exhibited high similarity of DEG functional responses after FL exposure, which clustered into inflammation, immune, and acute phase responses. These responses are known to be regulated by principle up-stream transcription factors, such as *IL1B*, with downstream support through middle regulators, *IL1A, TNF, IFNγ*, and other factors such as *NFkβ, STAT3, JUN*, and *RELA*. Within the skin dataset, subsets of these transcriptional regulators were observed to be up-modulated among all three organisms tested. For example, FL-modulated skin pathways in all three organisms contributed to an overall increase in the immune and inflammation responses, likely controlled by the *IL1B* upstream regulator that is differentially regulated in mouse (up-modulated 4.1-fold, *p*-value, 2.36E-05), medaka (up-modulated 6.7-fold, *p*-value 2.07E-67), and zebrafish (up-modulated 2.7-fold, *p*-value 1.73E-09) (Tables S3a–c). The numbers of skin DEGs identified after FL exposure was robust enough to allow analyses of predicted upstream regulators of the genetic response, as shown in Figure 4. Here we see remarkable similarity in regulators of the skin FL response among all three animals. In medaka and zebrafish skin, the upstream regulators, such as *IL1B* and *TNF*, and middle regulators (*SMAD3, RELA, STAT3, NFkβ, EGR1, JUN*) were part of the DEG dataset, whereas in the mouse, the role of these regulators was predicted by IPA based on the direction, levels, and numbers of DEGs modulated in the dataset (Figure 4).

### 3.2. Genetic Response of Brain to Fluorescent Light

The brain FL-mediated response of adult zebrafish was considerably lower than in skin (78 modulated genes compared to 364 genes in skin; Table S4a), and only 11% (9 genes) of the DEGs were shared between brain and skin (Figure 1, top). The FL modulated genes in zebrafish brain clustered into four significant canonical pathways (Table 3, top), all of which were associated with mounting inflammatory, immune and acute phase responses (Table 3).

The top regulator of the zebrafish brain FL responsive DEG dataset was *OSM* (z-score 2.77, *p*-value 4.97E-03), known to be controlled by *TNF* (2.38, 9.85E-03). Together this indicates an FL-induced up-modulation in the immune and inflammation responses, as observed in the skin, but via use of unique genes and pathways.

In medaka brain, 587 genes became differentially expressed after FL exposure and these genes were associated with 22 canonical pathways (20 up-modulated and two down-modulated, Table 3, middle). Sixteen of the 22 pathways (Table 3) were directly associated with inflammation, immune, and acute phase responses, with APR the top up-modulated pathway.

**Figure 5:**
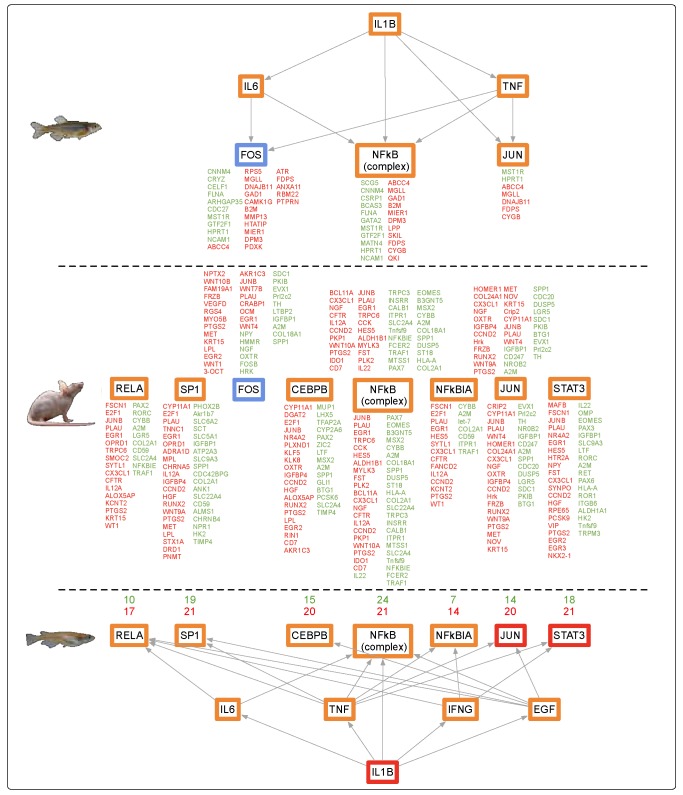
*IL1B* controls expression of many shared mid-level regulators in the zebrafish (top), medaka (bottom), and mouse (middle) brain datasets, including the key immune and inflammation APR regulator *TNF*. Genes in red and green are modulated DEGs, and genes in orange and blue are predicted by IPA to be up and down, respectively. Due to the robust dataset in medaka, the number of genes up- and down-modulated are listed in red and green, respectively.

FL exposure modulated substantially more genes (1172 DEGs) in mouse brain than in any other organ in mouse or zebrafish. Like zebrafish, the mouse brain showed an organ specific gene set expressed after FL, with only 9% (nine genes) of the DEGs in mouse brain shared with induced DEGs in mouse skin (Figure 1, middle). While many more genes were differentially modulated in the mouse brain, compared to mouse skin, or zebrafish and medaka brain, the same pathway responses were observed (Table 3 and Figure 5). Of the modulated pathways, APR and long-term potentiation were shared between mouse, zebrafish, and medaka brain samples after FL. The DEGs predicted the same upstream regulators, and direction of regulation, between zebrafish, medaka, and mouse brain following FL exposure (Figure 5).

### 3.3. Genetic Response of Liver to Fluorescent Light

Following FL exposure, the zebrafish liver showed only 64 DEGs (Table S4a), sharing only 9% (six genes) of the response with skin and 3% (two genes) with brain (Figure 1, left). There were five significant canonical pathways up-modulated in liver and four were associated with inflammation, immune, and acute phase responses (Table 4).

Although liver was expected to be the most indirect FL light-receiving organ tested, the response in zebrafish liver very closely mimicked skin, the most direct light receiving organ. The most significantly up-modulated pathway was APR (Table 4, 18 genes, z-score = 2.5), with the classical complement system closely following (9 genes, z-score 2.3). Like skin, all modulated pathways may be controlled by *TNF* (z-score 2.89, *p*-value 7.85E-03) to up-regulate transcription of genes involved with the immune and inflammation responses (Table 4).

In medaka, analysis showed a robust set of 3464 DEGs in liver after FL exposure. This large DEG set corresponded to predicted modulation of 33 canonical pathways (22 up-modulated and 11 down-modulated, Table 4, middle).

In medaka liver, as with skin and brain, the top modulated pathway was APR. Unlike zebrafish, each organ in medaka shared approximately 40% DEG gene identity with the other organs tested (Figure 1, right; 44% between skin and liver, 40% between skin and brain and 47% between brain and liver).

Following FL exposure, mouse liver differentially modulated 166 genes (≈37% more genes than zebrafish liver), which are expected to down regulate nine canonical pathways (Table S4c, Table 4). The most differentially expressed and suppressed pathways in the mouse liver dataset was the IL-6 signaling pathway (−4.0 z-score, 26 genes) and the APR (z-score −4.0, 19 genes). These canonical pathways were oppositely modulated in direction between mouse liver and mouse skin or brain, as well as opposite compared to all organs in zebrafish and medaka.

**Figure 6:**
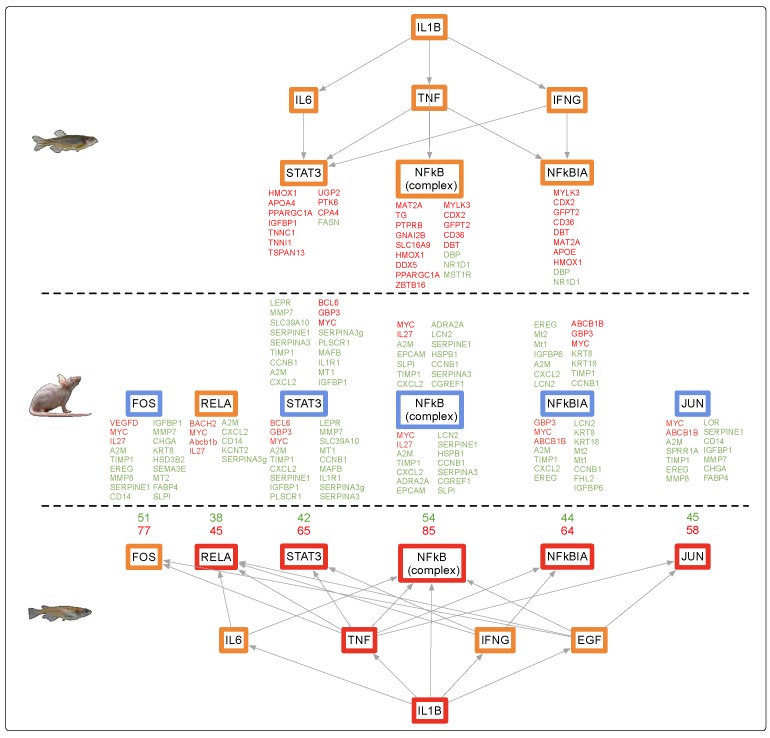
*IL1B* controls expression of many shared mid-level regulators in the zebrafish (top), medaka (bottom), and mouse (middle) liver datasets, including the key immune and inflammation APR regulator *TNF*. Genes in red and green are modulated DEGs, and genes in orange and blue are predicted by IPA to be up and down, respectively. Due to the robust dataset in medaka, the number of genes up and down-modulated are listed in red and green, respectively.

The mouse liver exhibited modulation of the same pathways and upstream regulators observed upon FL exposure in skin and brain (Figure 6). In addition, mouse, medaka, and zebrafish liver appeared all tightly regulated by the same core set of upstream regulators, including *TNF*, *IL1B, IFNγ, IL-6, NFkβ*, and *STAT3*; however, all of these genes were modulated in the opposite direction in mouse liver compared to the two fishes (Figure 6).

Of the significantly modulated pathways, three were shared between mouse liver and skin (granulocyte adhesion and diapedesis, IL-1 mediated inhibition of RXR, and LXR/RXR activation), one between liver and brain (IL-6 signaling), and one between all three organs (APR); however, all were modulated in the opposite direction when compared to mouse liver. In addition, two pathways (APR and IL-1 mediated inhibition of RXR) were shared between mouse and zebrafish liver samples, and three pathways (APR, LXR/RXR activation, and IL-1 mediated inhibition of RXR), between medaka and mouse liver, though all pathways were modulated in the opposite direction in the mouse.

### 3.4. Conservation Among Fluorescent Light Responses

When comparing the canonical pathways that were modulated in each organ for all three vertebrates, the majority of the pathways were associated with induction of cellular inflammation and immune responses. For example, in zebrafish brain, all four canonical pathways lead to an increase in the immune and inflammation response, in mouse brain 70% (all but six pathways) were involved, and in medaka brain 64% (14 pathways) of the pathways aided in the increased modulation of immune and inflammatory responses. An overlap analysis of the functional categories modulated in each brain sample indicated that although mice modulated over 15 times more genes following FL exposure than zebrafish, and twice as many as medaka, the predicted functional effects were highly conserved among all three vertebrates.

**Figure 7:**
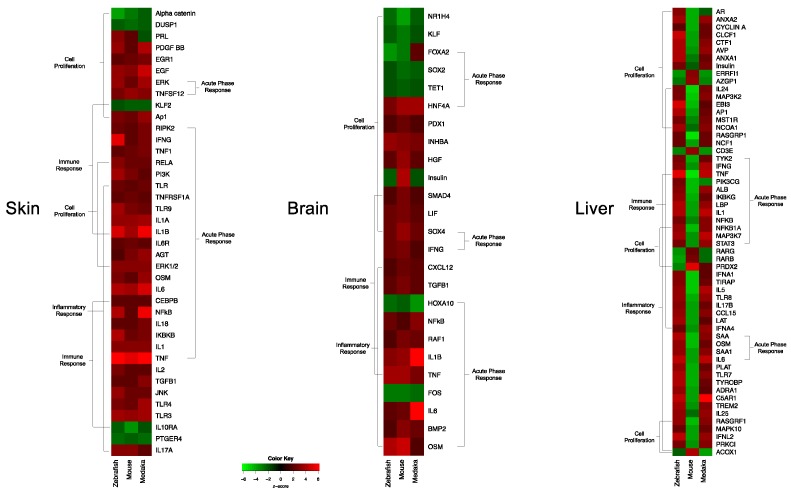
Zebrafish, medaka, and mouse shared up-stream regulators differentially modulated (z-score ± 2) in skin (left), brain (center) and liver (right) following FL exposure. Red represents regulators that are up-modulated in response to FL, and green represents down-modulation as predicted by IPA. Medaka and mouse have one regulator in liver, *PRDX2*, that is shared in the same direction.

Such transcriptional response similarities after exposure to FL, in three organs of three highly diverged vertebrates, suggested shared regulatory circuitry. Analysis of expression for upstream regulators derived from the data itself, or predicted by IPA based upon the DEG sets, showed remarkable similarity for all organs. Figure 7 shows the expression patterns for regulators of cell proliferation, acute phase response, immune response, and inflammatory response for all three animals and organs. It was clear these upstream regulators all tracked together after FL exposure, for both fish species and the mouse in skin and brain (Figure 7, skin and brain). A very similar pattern of effect within the same functional classes of regulators was observed for liver, with the notable exception that mouse liver upstream regulators were modulated in the opposite direction compared to the fishes (Figure 7, liver).

The trends in zebrafish, medaka, and mouse brain observed following FL exposure could be traced from the upstream regulators to the functional effects (Figure 4, Figure 5 and Figure 6). A direct line could be drawn from the up-modulation of *TNF* (2.38 zebrafish, 3.39 medaka, and 2.63 mouse) through the uniquely expressed gene sets that were regulated by up-modulation of *TNF* (11 zebrafish, 112 medaka, and 129 mouse), leading to the up-modulation of the TH1 immune response of T lymphocytes and cell proliferation, as well as the suppression of apoptosis in zebrafish and mouse. This highly shared response was also observed in zebrafish, medaka, and mouse skin and liver, though in the opposite direction for the mouse liver. In the liver, 33%, or 93 regulators, of the zebrafish response were shared by identity with medaka and mouse. Of these 93 shared regulators, 62% (58 regulators) were involved with an immune or inflammation response (Figure 7, liver). The immune and inflammation response in all organs from all three vertebrates could be traced through the top modulated regulator, *TNF*, directly to *IL1B* (Figure 4, Figure 5 and Figure 6). Many of the other regulators involved were directly linked to the APR, which was the only shared canonical pathway modulated in all three organs and all three animals (Figure 2).

## 4. Discussion

**Figure 8:**
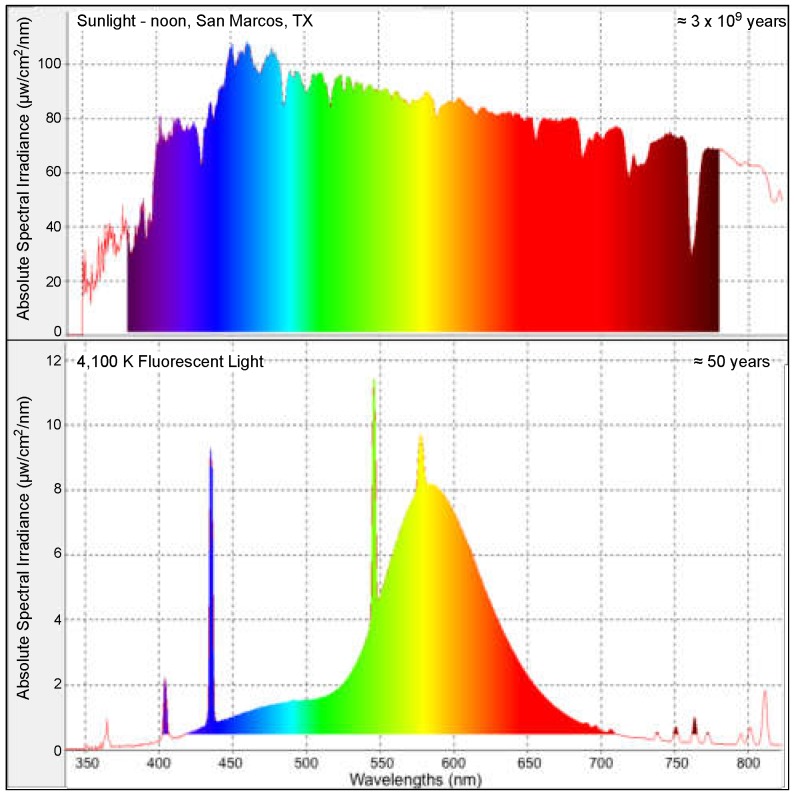
Examples of typical emission spectra from the sun (top) and 4100 K FL, (bottom).

Fluorescent light (FL) has only been commonly utilized as an inexpensive artificial light source for homes, office buildings, and research facilities since the late 1960′s. Increasing use of FL in animal and human environments has led to longer daily exposures to FL sources, yet careful studies of potential genetic consequences that may result from increased FL exposure have not been performed. Since the complex solar spectrum concurrently provides nearly all visible spectrum wavelengths in similar intensities (Figure 8), organisms had the opportunity to conscript each wavelength as cues to regulate selected genetic pathways, allowing interaction with their environment. Thus, significantly narrowing the complexity of available light wavelengths, as occurs under FL and other types of artificial light, may not incite the total genetic response fine-tuned over evolutionary history to the solar spectrum.

Herein, we extend previous observations of fish skin to investigation of FL-induced genetic effects within internal organs (i.e., brain and liver). We provide head-to-head comparisons of FL-induced modulation of gene expression among two fish species (i.e., zebrafish and medaka) with each other, and with the hairless mouse (*Mus musculus*). The fish species utilized (medaka and zebrafish) are diurnal vertebrates originally derived from Japan and India, respectively, with an estimated divergence of ≈115 My [31,32,33]. The mouse, a nocturnal rodent, has an estimated ≈450 My of divergence from the common ancestor that led to the fishes [34,35]. All animals were similarly exposed to FL (4100 K), and the transcriptional response in skin, brain, and liver organs compared after processing of RNA-Seq data. Our findings suggest the primary response to FL is extraordinarily well-conserved among these three highly divergent species. This suggests the gene expression changes that occur after light exposure may be due to ancient genetic circuitry that has remained embedded within the vertebrate genome.

### 4.1. Alternate Pathway Analysis and Cross Validation

As a secondary confirmation of the pathway and functional annotation categories identified by IPA, an independent enrichment analysis using Consensus PathDB was performed (http://consensuspathdb.org, [36,37]). Consensus PathDB uses a collection of published interaction databases including biochemical pathways, protein interactions, genetic interaction signaling, metabolism, gene regulation, and drug-target interactions, and an enrichment analysis to determine what biological functions are over-represented (enrichment *p*-value < 0.05) in a dataset. Consensus PathDB’s functional enrichment analyses showed genes related to innate immune response, immune system development, interferon production, cytokine production, and wound healing response, as well as oxidation-reduction process, response to oxygen containing compounds, and response to oxidative stress are over-represented (*p* < 0.05) among the genes modulated by FL exposure. This observation supports the results obtained from IPA in each of the zebrafish, medaka, and mouse organs (Figures S1–S3). Other functions that were enriched following Consensus PathDB analysis included cell–cell signaling, defense and stress response signaling, apoptosis, cell cycle regulation, lipid metabolism, cellular transport, and circadian rhythm regulation.

As bioinformatics analyses showed that inflammation and immune related genes dominate the genetic response incited by FL, we hypothesized that gene expression pattern change after FL exposure should be similar to that after known treatments that lead to inflammation and infection. We compared the FL-incited DEGs to literature-reported organism and organ-matched transcriptomic data associated with an inflammatory condition, immune response, and acute phase response [38,39,40]. Mouse skin FL-incited DEGs were compared to DEGs modulated by Imiquimod (IMQ), a compound that is used to induce psoriasis-like skin inflammation [41,42]; mouse liver FL-incited DEGs were compared to DEGs modulated by Lipopolysaccharide (LPS) challenge; and zebrafish liver FL-incited DEGs were compared to DEGs modulated by *Edwardsiella tarda* vaccination. In mouse skin and zebrafish liver, the majority (100% in mouse skin and 63% in zebrafish liver) of DEGs exhibited consistent direction of modulation from FL exposure or from treatment performed by each response based on the published data. These observations match the functional analyses performed using the FL exposure dataset. In contrast, mouse liver showed opposite modulation in all but one gene between FL and LPS challenge, suggesting FL exposure led to an opposite genetic response compared to LPS induction. This result indicates the immune response is repressed by FL, while induced by LPS challenge administered over a six-day period (Figure S4).

### 4.2. Fluorescent Light Genetic Response Conservation in the Skin of Vertebrates

We show that upon FL exposure of the intact animal, the skin of both fishes and mice exhibited APR pathway induction, as well as species-specific APR sub-pathways, as part of an overall inflammation and immune response. For example, in zebrafish skin, Oncostatin M is induced by FL (Table 2, z-score 3.19, 10 genes). This APR sub-pathway is known to play a role in inflammation, promote production of *IL-6* in epithelial cells, as well as regulate many key APR proteins [43,44,45]. In addition, zebrafish showed activation of the nitric oxide and oxidative stress sub-pathway (Table 2), suggesting that after FL, the skin has the perception of reactive oxygen species (ROS) and potential oxidative damage [46]. This may occur due to the light exposure itself; however, the lower wavelengths (i.e., UVA) that are expected to lead to ROS production are a very minor component of the FL spectrum (Figure 8). Alternatively, activation of nitric oxide pathways may be an offshoot of FL effect on cell division, which is generally reduced when entering the circadian light cycle [25].

Medaka exhibited a strong APR response that was more robust than observed in zebrafish or the mouse (Table 2, z-score 3.47, 45 genes). Unique medaka FL-induced APR sub-pathways included RhoA signaling, Eicosanoid signaling, Ceramide signaling, p38MAPK signaling, and others. Induction of these pathways indicated medaka skin has perceived oxidative stress (i.e., p38MAPK and others), likely from initial cytokine induction by the APR, and has begun to reorganize cellular structure in response (i.e., RhoA, Eicosanoid, and Ceramide signaling pathways).

Upon FL exposure, the mouse skin not only exhibited induced components of the APR response, but also showed up-modulation of immune cell activation (Table 2, leukocyte extravasation, hepatic stellate cell activation, granulocyte adhesion, agranulocyte adhesion, etc.). In support of this cell-based response, the mouse NanoString PanAm panel, used to validate the RNA-Seq data, is able to assess changes in cell populations. In FL exposed mice, T-cells increased two-fold in skin and 2.3-fold in liver. In addition, CD45, a common leukocyte antigen, increased 1.3-fold, 1.2-fold, and 1.4-fold in skin, liver, and brain, respectively, following FL exposure, while macrophage increased 1.4- and 1.5-fold in both skin and liver. Taken together with the analyses of altered gene expression, these data suggest the mouse organs tested all up-modulate a cellular inflammation and immune response.

In skin, four canonical pathways were shared between zebrafish, medaka, and mouse, and each of these has considerable genetic overlap. For example, 12 genes were differentially modulated in the triggering receptor expressed on myeloid cells *1* signaling pathway (TREM1, Figure 3) for zebrafish and mouse, while medaka modulated 13 TREM1 genes. TREM1 is a key signaling pathway in the inflammatory response due to its role in *TNF*, *IL-6*, *IL-8*, *JAK2*, and *ERK1/2* activation, *JAK2* and *ERK1/2* activation leads to increased expression of *NFkβ*, *STAT3*, and *STAT5*. Many of these cytokines were shared among all three organisms analyzed, including the upstream regulators *STAT3*, *TNF*, and *IL1B* (Tables S3a–c, Figure 4, Figure 5, Figure 6 and Figure 7). These transcription factors in turn activate an inflammatory response [47,48].

Overall, the primary response of FL-exposed skin indicated zebrafish, medaka, and mouse all increase cellular functions to activate an immune and inflammation response that is highly conserved. The highest up-stream regulator for zebrafish, medaka, and mouse skin was *TNF* (6.16-, 5.88-, and 5.39-fold, respectively) followed closely by *IL1B* (5.23-, 5.58-, and 4.51-fold, respectively). *TNF* and *IL1B* both increase pathway modulation of oncostatin M and TREM1 before activating the APR signaling pathway. Therefore, it is not surprising that the down-stream effects (increased inflammation and immune response) are conserved, since the upstream regulators are so tightly correlated among these three divergent biomedical research models.

### 4.3. Fluorescent Light Genetic Response Conservation in the Brain of Vertebrates

The top dysregulated canonical pathway in the zebrafish brain, also up-modulated in the brain of mice, but down-modulated in medaka, was long-term potentiation. This pathway is known to increase and strengthen the synapses in the brain based on the activity of the animal. It is used primarily by animals to increase synaptic plasticity and; therefore, decrease the amount of signal necessary to generate a response [49]. In addition, to long-term potentiation, zebrafish brain induced three canonical pathways, coagulation system, intrinsic prothrombin activation, and the APR signaling pathway. Along with the up-stream regulators, up-modulation of these pathways also suggested the brain is increasing immune and inflammation responses, as well as making increased synaptic memories so the organism can properly respond quickly to the same physical stimulus in the future. In this way, the brain linked the physical light response through a conserved neuronal transcriptional response, leading to genetic induction of gene sets that produced an inflammation and immune response.

The suppression of long-term potentiation (−3.32-fold, 34 genes) in medaka brain was perplexing, given the high genetic conservation observed in other pathways after FL exposure. Overall, the medaka brain showed a robust genetic response to FL; including APR as the top activated pathway (3.67 z-score, 20 genes) and activation of a large list of APR sub-pathways (IL-6 signaling, JAK/STAT, GNRH signaling, hypoxia signaling, IL-2 signaling, PPAR signaling, etc.). The extent of this list, compared to zebrafish and the mouse, suggested medaka brain had developed a more mature response (i.e., forward gene expression movement into a full response) in the six hrs between FL exposure and RNA isolation, while the other two animals may require a longer time period to fully execute APR and APR sub-pathway activation. Medaka may have activated the APR at a lower threshold and more rapidly than zebrafish or mice, and thus, early steps in pathway activation in response to FL (e.g., long term potentiation) may have already peaked and are no longer needed to induce downstream cytokines. In any case, the overall response of medaka brain to FL involved the same conserved pattern of inflammation and immune function activation as presented by the other two animals, perhaps just later in the process.

The FL response in mouse brain was very similar to the medaka skin response (Table 2 and Table 3). Both mouse brain and medaka skin show up-regulation of APR, Gαs 12/13 signaling, RhoA signaling, IL-6 signaling, ILK signaling, VEGF signaling, and B cell receptor signaling. Thus, as with medaka skin, it appears the mouse brain has begun to reorganize cell structures and affect cell cycle progression in anticipation of real or perceived ROS damage.

Suppression of the glutamate receptor signaling pathway (GRS) in the mouse brain after FL exposure was interesting. GRS has two primary functions within neural cells. One is cell communication associated with memory formation [50,51], by adjusting the number of ionotropic glutamate receptors to regulate long term potentiation [52,53]. Secondly, increased activity of the GRS pathway can suppress *TNF-* and ROS-mediated cascades that lead to inflammation and cell death in response to cellular oxidative damage [54,55]. Therefore, the observed suppression of GRS following FL exposure (−2.00-fold, 11 genes) in mouse brain likely serves to increase *TNF* cytokine interaction, activating caspase dependent apoptosis, inflammation, and cell death in neuronal cells. This was supported by the increased expression of ILK signaling (2.03-fold).

### 4.4. Fluorescent Light Genetic Response Conservation in the Liver of Vertebrates

In zebrafish liver, the APR and classical complement pathways, one of the first steps in the inflammation cascade, were upregulated (Table 4, 2.3-fold, 9 genes). This agrees with the FL response in other organs as the perception of eminent oxidative stress, with the resulting induction of genes and pathways to mount an inflammation and immune response. Again, the medaka liver exhibited a long list of activated APR sub-pathways, indicating a fuller or more mature inflammation and immune response then observed for zebrafish liver.

Following FL exposure, the mouse liver was the only organ, among the organs tested, predicted to down-modulate upstream regulators *IL1B, TNF*, and *TGFβ* based on the DEG sets. This indicated the mouse liver was predicted to suppress inflammation and immune function in response to FL exposure. Key pathways, including *IL-6* (z-score −4.0, 26 genes), APR pathway (z-score −4.0, 19 genes), and STAT3 (z-score −3.0, 7 genes), were down-modulated in mouse liver, but up-modulated in zebrafish, medaka, and all other mouse organs tested. Mice are nocturnal animals and it is reported that metabolic processing and cellular turnover is reversed from diurnal animals, such as humans and fish [56,57,58,59,60]. Therefore, RNA isolated at the onset of the light cycle for both fish and mice reflect their entry into active and inactive periods, respectively. After FL exposure, transcriptional activity of the upstream regulators in zebrafish and mouse liver were exactly opposite; except for one regulator, *PRDX2*, that was up-regulated in both medaka and mice but suppressed in the zebrafish liver samples. *PRDX2* is an antioxidant enzyme used to reduce hydrogen peroxides and protect cells from oxidative damage. The increase in *PRDX2* gene expression in both medaka and mouse supported cellular perception of oxidative stress.

The opposite FL response in the liver of mice, compared to the other organs or fishes, is consistent with reports indicating liver metabolism follows an animal’s “activity time” [57,61]. Metabolic processes in particular are reversed in nocturnal animals and more closely correspond to feeding times, which generally occur during the animal’s active period. For example, insulin is reported to oscillate in liver out of phase with the circadian light cycle, regardless of stimulation [62,63]. Our RNA-Seq data support insulin as an upstream regulator (Tables S5a–c, mouse liver insulin −2.02-fold, zebrafish 2.56-fold, and medaka 2.37-fold). It has been shown that feeding nocturnal animals during their natural resting period may result in diminished liver metabolism, leading to obesity, compared to the animals fed during their active periods [56,58,62,63]. Thus, although the mouse liver may perceive light-induced damage, the metabolic background at entry into the rodent’s sleep cycle may have prevented the “normal” light cycle genetic response observed in the other organs (brain and skin), as well as all organs in the diurnal fishes.

### 4.5. Evolutionary Comparisons Highlighting Transcriptional Activation of the APR

The question of how FL serves to incite the APR, with the inflammation and immune responses, is of interest. APR is known to have multiple modes of initiation including local trauma, bacterial or viral infection, oxidative cellular stress, etc. [28,29,64]. Interestingly, neural signals initiated by opsonins initiate a neoplasia inflammatory response in the brain, that quickly signals to local pro-inflammatory cytokines *IL1B* and *TNF* throughout the body [65,66]. This in turn activates neutrophils that signal liver hepatocytes to modulate protein synthesis and increase APR enzymes throughout the body via transport in the blood stream, to increase inflammation and minimize oxidative damage [27,67,68,69].

Each response in the respective organs is controlled through the induction of key regulators *TNF* and *IL1B* (Figure 4, Figure 5 and Figure 6) that modulate 72%–88% of the zebrafish DEGs, 65%–74% of the medaka DEGs, and 61%–79% of mouse DEGs. Following gene modulation, an innate inflammatory response is up-modulated in skin and brain of all three animals, with over 30% of the functional categories being shared in any two-organ comparison. The functional categories modulated following this response can be grouped into four primary categories: immune cell trafficking, inflammatory response, cellular movement, and cell to cell signaling. Each of these categories is a branch of the APR signaling pathway, and together serve to stimulate the immune and inflammation responses to compensate for the perception of increased oxidative cellular stress.

It seems likely the intense light exposure (35 and 52.5 kJ/m^2^) over a short time (40–60 min) may have saturated the normal light response, changing the light cycle for the animals in a matter of minutes, rather then gradually, as would be expected in more natural conditions. The evolutionary history may have entrained the genetic response to intense light (i.e., noon) to include protection against ROS, since anytime light was received under the solar spectrum, UVA, and shorter wavelengths would have been a major component. This explains why UV specific DNA photorepair proteins (i.e., cyclobutene pyrimidine dimers and 6–4 photoproduct DNA photolyases) become transcriptionally induced upon exposure to visible light, and not UV light for which their activity is needed. Animals simply never saw visible light without a UV component until they became housed in artificially-lit facilities. Therefore, it may not be surprising that exposure to the 4100 K FL spectrum leads to inflammation and immune responses, even though the UV wavelengths emitted, that induce ROS, are a very small fraction of the light received (Figure 8). Recently, we reported that *Xiphophorus* exposed to specific light wavelengths induce and suppress select genetic pathways in a wavelength-specific manner [3]. In addition, different wavelengths were identified that could induce, or suppress, the same genetic pathways. This supports the concept that over evolutionary time, animals may have conscripted specific wavelengths of light for select genetic regulatory responses, and the summation of all solar wavelengths may be needed to properly respond to the environment. We also determined similar wavelength specific genetic responses occur in both zebrafish and medaka (in preparation). Given the high conservation of the FL response in fish and mice shown here, it will be interesting to determine whether mammals have retained wavelength-specific genetic regulation, and these experiments are currently underway.

## 5. Conclusions

We present results that assessed changes in gene expression patterns due to FL exposure in zebrafish and medaka fishes, and in hairless mice. Following FL exposure, RNA from skin, brain, and liver was utilized for RNA-Seq, and gene expression was validated with NanoString nCounter assays. Differentially expressed genes (DEGs), due to the FL exposure, were utilized in functional analyses to identify FL-affected biological pathways for comparison.

All organs, in all three animals, respond to FL by modulating pathways leading to inflammation and immune responses. These conserved genetic responses involved induction of the acute phase response (APR) in all organs of the fish species, and mouse skin and brain. The pathways affected by FL are regulated primarily by *TNF* and *IL1B* and are predicted to induce APR, leading to inflammation and immune responses. The only exception was the mouse liver, that showed suppression in the same APR pathways that were activated in the other organs examined. APR suppression in the mouse liver may be due to a nocturnal metabolism keeping the liver out of phase with FL exposure. Collectively, the conserved FL genetic response in both fishes and mice appear due to cellular perception of oxidative stress.

These data suggest the primary response to FL is extraordinarily well-conserved among highly divergent species, representing both diurnal and nocturnal lifestyles, and; therefore, deeply embedded within the vertebrate genome.

## Figures and Tables

**Table 1:** Differentially expressed genes for fluorescent light (FL) exposed samples. Total modulated genes are the output file from EdgeR (column 2) that had a log_2_ (fold change) ≥ |2.0| and a (*p*-adj < 0.05). All fish Ensembl IDs were converted to Human Genome Organization (HUGO) IDs (column 5) for Ingenuity Pathway Analysis (IPA) analysis and direct comparison of the zebrafish, medaka and mouse. HUGO IDs were then imported and mapped by Qiagen’s IPA software for functional and pathway analysis (column 6). Mouse Ensembl IDs were directly imported into IPA for functional and pathway analysis.

Species	Organ	Total Modulated	Up-Modulated	Down-Modulated	HUGO IDs	Mapped by IPA
Zebrafish	Skin	523	336	187	482	364
	Brain	94	49	45	89	78
	Liver	83	56	27	79	64
Medaka	Skin	2304	1229	1075	2298	2284
	Brain	592	433	159	589	587
	Liver	3492	1684	1808	3483	3464
Mouse	Skin	107	49	58	N/A	102
	Brain	1174	699	475	N/A	1172
	Liver	182	51	131	N/A	166

**Table 2:** Modulated canonical pathways were predicted using IPA for zebrafish (top), medaka (middle), and mouse (bottom) skin. All pathways with a z-score ≥ |2|, *p*-value < 0.05, and five or more genes were included in the analysis. The column on the far right indicates if the pathway is involved in an immune (Im), inflammatory (If), acute phase (AP), cellular proliferation (CP), or cellular signaling (CS) response.

**Zebrafish Skin**				
**Ingenuity Canonical Pathways**	**z-score**	***p*-value**	**Gene**	**Category**
Acute Phase Response Signaling	4.12	4.27E-02	9	Im, If, AP, CP
Production of Nitric Oxide and ROS in Macrophages	4.11	2.29E-02	11	Im, AP
Oncostatin M Signaling	3.19	3.09E-03	10	Im, If, AP, CP
Leukocyte Extravasation Signaling	3.07	2.88E-03	14	Im, If
TREM1 Signaling	3.00	2.69E-02	12	Im, If, AP
mTOR Signaling	2.90	2.09E-02	12	CP, CS
Complement System	2.71	2.02E-02	6	Im, If, AP
IL-6 Signaling	2.45	2.96E-02	11	Im, If, AP
B Cell Receptor Signaling	2.00	3.98E-02	10	Im, AP
ILK Signaling	2.00	4.27E-03	13	Im, If, AP, CP
Nitric Oxide Signaling in the Cardiovascular System	2.00	4.16E-02	6	If
NRF2-mediated Oxidative Stress Response	2.00	1.02E-02	9	If, AP
Role of NFAT in Regulation of the Immune Response	2.00	4.33E-02	6	Im, AP
Thrombin Signaling	2.00	1.28E-02	9	If, AP
Gα12/13 Signaling	−2.00	4.17E-03	10	If, AP, CP, CS
PPARα/RXRα Activation	−2.37	2.46E-01	7	If, AP
Cardiac Hypertrophy Signaling	−2.87	7.76E-03	14	If, AP, CP, CS
eNOS Signaling	−2.97	4.79E-02	8	If, CP
CDK5 Signaling	−3.56	2.09E-03	9	If, AP, CP
Calcium Signaling	−3.58	7.94E-12	26	CP, CS
Actin Cytoskeleton Signaling	−2.47	2.40E-03	15	AP, CP, CM, CS
**Medaka Skin**				
**Ingenuity Canonical Pathways**	**z-score**	***p*-value**	**Genes**	
Acute Phase Response Signaling	3.67	3.39E-09	45	Im, If, AP, CP
Gα12/13 Signaling	2.79	4.68E-03	24	If, AP, CP
Pancreatic Adenocarcinoma Signaling	2.79	1.10E-05	29	Im, AP, CP
RhoA Signaling	2.79	4.27E-03	23	If, CP, CM
Eicosanoid Signaling	2.73	9.33E-03	14	If
IL-6 Signaling	2.68	1.82E-05	30	Im, If, AP
ILK Signaling	2.49	7.59E-05	39	Im, If, AP, CP
TREM1 Signaling	2.43	5.01E-02	13	Im, If, AP
Endothelin-1 Signaling	2.41	5.75E-04	35	AP, CP, CM
Ceramide Signaling	2.37	7.76E-05	23	If, AP, CP
ERK5 Signaling	2.22	1.35E-02	13	CP
NGF Signaling	2.20	5.25E-03	22	AP, CP
p38 MAPK Signaling	2.20	2.45E-03	23	Im, If
Activation of IRF by Cytosolic Pattern Recogn. Recep.	2.16	2.05E-02	9	Im, AP
MIF Regulation of Innate Immunity	2.16	2.51E-02	9	Im, AP
Cardiac β-adrenergic Signaling	2.10	1.45E-02	23	CS
VEGF Signaling	2.10	7.41E-02	16	AP, CP, CM
B Cell Receptor Signaling	2.06	1.86E-03	33	Im, AP
NANOG in Mammalian Embryonic Stem Cell Pluripotency	2.00	1.51E-04	27	AP, CP
ATM Signaling	−2.00	3.98E-02	14	If, CP
Wnt/GSK-3β Signaling in the Pathos of Influenza	−2.00	1.26E-02	15	Im
NFAT in Cardiac Hypertrophy	−2.11	6.31E-03	32	Im, AP
PPARα/RXRα Activation	−2.68	1.70E-02	17	If, AP
Antioxidant Action of Vitamin C	−2.82	4.17E-02	17	If, AP, CP
**Mouse Skin**				
**Ingenuity Canonical Pathways**	**z-score**	***p*-value**	**Genes**	
TREM1 Signaling	4.20	2.00E-05	12	Im, If, AP
Role of IL-17F Inflammatory	4.00	3.98E-05	5	If
Leukocyte Extravasation Signaling	3.00	1.26E-02	5	Im, If
Hepatic Stellate Cell Activation	2.30	1.58E-05	7	If
AMPK Signaling	2.20	1.00E-02	5	CP
Granulocyte Adhesion and Diapedesis	2.20	2.00E-11	12	If
IL-1 Mediated Inhibition of RXR Function	2.20	1.58E-02	5	Im, If, AP
PPARα/RXRα Activation	2.20	7.94E-03	5	If, AP
Acute Phase Response	2.10	3.98E-03	17	Im, If, AP, CP
Inhibition of Matrix Metalloproteases	2.10	2.51E-05	5	CM
LXR/RXR Activation	2.10	2.00E-03	5	Im, If
Agranulocyte Adhesion and Diapedesis	2.00	1.00E-08	10	Im, If

Abbreviations: Reactive oxygen species (ROS), Triggering receptor expressed on myeloid cells 1 (TREM1), Mammalian target of rapamycin (mTOR), Interleukin 6 (IL-6), Integrin-linked kinase (ILK), Nuclear factor erythroid 2-related factor 2 (NRF2), Nuclear factor of activated T-cells (NFAT), Peroxisome proliferator activated receptor alpha (PPARα), Retinoid X receptor alpha (RXRα), Endothelial nitric oxide synthase (eNOS), Cyclin dependent kinase 5 (CDK5), Ras homolog gene family member A (RhoA), Extracellular signal-regulated kinase 5 (ERK5), Nerve growth factor (NGF), Mitogen activated protein kinase (MAPK), Interferon regulatory factor (IRF), Migration inhibitory factor (MIF), Vascular endothelial growth factor (VEGF), homeobox transcription factor Nanog (NANOG), Ataxia-telangiectasia mutated (ATM), Liver X receptor alpha (LXR), Wingless integrated (Wnt), Glycogen synthase kinase 3 beta (GSK-3 β), Adenosine monophosphate activated protein kinase (AMPK), Interleukin-1 (IL-1).

**Table 3:** Modulated canonical pathways were predicted using IPA for zebrafish (top), medaka (middle), and mouse (bottom) brain. All pathways with a z-score ≥ |2|, *p*-value < 0.05, and five or more genes were included in the analysis. The column on the far right indicates if the pathway is involved an immune (Im), inflammatory (If), acute phase (AP), cellular proliferation (CP), or cellular signaling (CS) response.

Zebrafish Brain				
Ingenuity Canonical Pathways	z-score	*p*-value	Gene	Category
Synaptic Long-Term Potentiation	2.60	8.71E-03	13	Im, If, AP, CP
Intrinsic Prothrombin Activation Pathway	2.20	1.17E-08	6	If
Coagulation System	2.10	1.66E-08	6	If
Acute Phase Response	2.00	7.24E-04	17	Im, If, AP, CP
**Medaka Brain**				
Acute Phase Response Signaling	3.67	3.98E-08	20	Im, If, AP, CP
Endothelin-1 Signaling	3.36	2.00E-04	15	AP, CP, CM
B Cell Receptor Signaling	2.71	1.29E-02	11	Im, AP
Prolactin Signaling	2.65	1.91E-03	8	Im, AP, CP
ERK/MAPK Signaling	2.50	1.17E-03	14	CP
IL-6 Signaling	2.50	4.47E-05	13	Im, If, AP
CREB Signaling in Neurons	2.45	3.02E-02	10	AP, CP, CS
JAK/Stat Signaling	2.45	2.57E-02	6	Im, AP, CP
Thrombopoietin Signaling	2.45	8.51E-03	6	If, AP, CP
P2Y Purigenic Receptor Signaling Pathway	2.33	1.02E-02	9	If, AP, CP
GNRH Signaling	2.33	8.91E-03	9	If, AP, CP, CM
Melanocyte Development and Pigmentation Signaling	2.24	4.57E-02	6	AP, CP, CS
ERK5 Signaling	2.24	2.88E-02	5	CP
Hypoxia Signaling in the Cardiovascular System	2.24	1.91E-03	7	If, CP
NFAT in Regulation of the Immune Response	2.12	1.29E-02	11	Im, AP
PKCθ Signaling in T Lymphocytes	2.12	2.82E-02	8	Im, AP
Ephrin Receptor Signaling	2.12	3.02E-03	12	CP, CM, CS
Chemokine Signaling	2.12	6.92E-04	8	If, AP, CP, CM
cAMP-mediated signaling	2.00	3.98E-04	16	If, AP, CS
IL-2 Signaling	2.00	3.02E-02	5	Im, AP, CP, CS
PPAR Signaling	−2.83	3.89E-03	8	Im, AP
Synaptic Long-Term Potentiation	−3.23	3.98E-04	34	Im, If, AP, CP
**Mouse Brain**				
Actin Cytoskeleton Signaling	3.50	1.66E-03	22	CP, CM
Gαs Signaling	3.40	2.57E-03	11	If, AP, CS
RhoA Signaling	3.10	1.82E-02	12	If, CS
VEGF Signaling	2.60	4.68E-02	10	CP, CM
Nitric Oxide Signaling	2.50	1.35E-03	11	If, CS
Cyclins and Cell Cycle Regulation	2.40	4.37E-02	8	CP
Gα12/13 Signaling	2.30	2.24E-02	13	If, AP, CS
Cholecystokinin/Gastrin-mediated Signaling	2.10	3.89E-03	10	AP, CP, CM
CXCR4 Signaling	2.10	2.09E-02	16	Im, AP, CP, CM
B Cell Receptor Signaling	2.10	3.16E-02	18	If, Im, AP, CM
Acute Phase Response Signaling	2.10	3.80E-02	19	If, Im, AP, CP
Gαq Signaling	2.10	1.45E-03	15	If, AP, CM, CS
IL-6 Signaling	2.00	4.79E-02	21	If, Im, AP
Synaptic Long-Term Potentiation	2.00	1.95E-03	12	If, AP, CS
ILK Signaling	2.00	8.32E-04	19	If, Im, AP, CP, CM
Tec Kinase Signaling	2.00	4.79E-02	10	AP, CP, CM
Calcium-induced T Lymphocyte Apoptosis	−2.00	1.70E-02	6	Im
Glutamate Receptor Signaling	−2.00	4.37E-06	11	If, CS
FLT3 Signaling in Hematopoietic Progenitor Cells	−2.00	4.90E-02	9	Im, AP, CP
BMP signaling pathway	−2.10	2.09E-03	8	AP, CP

Abbreviations: Janus kinase (JAK), Signal transducer and activator of transcription (STAT), Purinergic G protein-coupled receptors (P2Y), Gonadotropin-releasing hormone (GNRH), Protein kinase C theta (PKCθ), Cyclic adenosine monophosphate (cAMP), Interleukin 2 (IL-2), G protein alpha subunit (Gαs), Ras homolog gene family member A (RhoA), Guanine nucleotide-binding protein alpha 12 and 13 subunit (Gα12/13), G protein subunit q (Gαq), FMS-like tyrosine kinase 3 (FLT3), Bone morphogenetic protein (BMP).

**Table 4:** Modulated canonical pathways were predicted using IPA for zebrafish (top), medaka (middle), and mouse (bottom) liver. All pathways with a z-score ≥ |2|, *p*-value < 0.05, and five or more genes were included in the analysis. The column on the far right indicates if the pathway is involved an immune (Im), inflammatory (If), acute phase (AP), cellular proliferation (CP), or cellular signaling (CS) response.

**Zebrafish Liver**				
**Ingenuity Canonical Pathways**	**z-score**	***p*-value**	**Gene**	**Category**
Acute Phase Response	2.5	3.89E-02	18	Im, If, AP, CP
Complement Signaling	2.3	5.62E-03	9	Im, If, AP
IL-1 Mediated Inhibition of RXR	2.0	1.35E-02	22	Im, If, AP
ILK Signaling	2.2	4.79E-02	19	If, Im, AP, CP, CM
Calcium Signaling	2.0	2.24E-02	17	CP, CS
**Medaka Liver**				
	**z-score**	***p*-value**	**Gene #**	**Category**
Acute Phase Response Signaling	3.2	9.77E-07	53	Im, If, AP, CP
Tec Kinase Signaling	2.9	1.02E-02	39	AP, CP, CM
mTOR Signaling	2.8	1.32E-02	44	CP, CS
PEDF Signaling	2.7	2.40E-06	31	AP, CP, CM
Insulin Receptor Signaling	2.6	1.70E-06	45	If, AP, CP
IL-1 Signaling	2.5	1.82E-06	33	If, AP, CS
Renal Cell Carcinoma Signaling	2.4	6.76E-03	22	Im, AP, CP, CM
HMGB1 Signaling	2.4	7.08E-04	36	If, Im, AP
NANOG in Mammal Embry Stem Cell Pluripotency	2.2	4.57E-03	31	AP, CP
Type I Diabetes Mellitus Signaling	2.2	3.80E-02	25	If, Im, AP
Toll-like Receptor Signaling	2.2	2.04E-03	22	If, Im, AP
UVB-Induced MAPK Signaling	2.2	2.69E-02	17	If, AP, CS
CNTF Signaling	2.2	1.74E-02	17	Im, AP, CP, CS
Pancreatic Adenocarcinoma Signaling	2.2	5.25E-05	36	If, AP, CP
IL-1 Mediated Inhibition of RXR	2.1	3.31E-03	51	Im, If, AP
PI3K/AKT Signaling	2.1	4.68E-06	40	If, Im, AP, CP
IL-8 Signaling	2.1	5.25E-07	59	If, Im, AP
Macropinocytosis Signaling	2.1	2.69E-02	20	Im
CD28 Signaling in T Helper Cells	2.1	2.14E-03	34	Im, AP
ERK5 Signaling	2.0	1.74E-04	22	CP
G-βγ Signaling	2.0	2.14E-03	25	If, AP, CP
MIF Regulation of Innate Immunity	2.0	4.07E-02	11	Im, AP
Chemokine Signaling	−2.0	2.75E-03	21	If, AP, CP, CM
Ephrin B Signaling	−2.1	2.57E-04	24	AP, CP, CM
Phospholipase C Signaling	−2.1	2.04E-02	50	AP, CP, CM
LXR/RXR Activation	−2.2	2.40E-06	40	Im, If
p53 Signaling	−2.2	7.59E-03	28	CP
CREB Signaling in Neurons	−2.3	2.69E-04	48	AP, CP, CS
Wnt/Ca+ pathway	−2.3	3.02E-05	22	CP
Sonic Hedgehog Signaling	−2.4	1.45E-02	10	CP
Dopamine-DARPP32 Feedback in cAMP Signaling	−2.7	8.71E-05	45	CS
Basal Cell Carcinoma Signaling	−2.9	7.41E-03	20	CP, CM, CS
Calcium Signaling	−3.2	8.32E-04	45	CP, CS
**Mouse Liver**				
	**z-score**	***p*-value**	**Gene #**	**Category**
IL-1 Mediated Inhibition of RXR	−2.0	6.92E-04	23	Im, If, AP
LXR/RXR Activation	−2.0	8.51E-03	13	Im, If
VDR/RXR Activation	−2.0	1.48E-02	8	Im, CP
Granulocyte Adhesion and Diapedesis	−2.1	1.15E-03	17	If
Coagulation System	−2.1	1.58E-03	5	If
STAT3 Pathway	−2.3	1.26E-02	7	If, Im, AP
p38 MAPK Signaling	−3.0	7.59E-03	12	Im, If
Acute Phase Response Signaling IL-6 Signaling	−4.0 −4.0	2.57E-02 1.55E-03	19 26	I Im, If, AP, CP m, If, AP

*Abbreviations: Pigment epithelium-delivered factor (PEDF), High-mobility group box 1 protein (HMGB1), Ciliary neurotrophic factor (CNTF), Phosphoinositide 3-kinase (PI3K), Protein kinase B (AKT), Interleukin 8 (IL-8), cAMP response element-binding protein (CREB), Vitamin D response (VDR)*.

## Supplementary Materials

The following are available online at https://www.mdpi.com/2073-4425/10/4/271/s1, **Figure S1:** Differentially expressed genes (DEGs) from fluorescent light (FL)-induced skin of zebrafish, medaka, and mouse were imported into Consensus PathDB. Dot size represents the number of DEGs represented in that function and dot color represents statistical significance measured by the *p*-value (red is a lower *p*-value and white is a higher *p*-value). **Figure S2:** DEGs from FL-induced brain of zebrafish, medaka, and mouse were imported into Consensus PathDB. Dot size represents the number of DEGs represented in that function and dot color represents statistical significance measured by the *p*-value (red is a lower *p*-value and white is a higher *p*-value). **Figure S3:** DEGs from FL-induced liver of zebrafish, medaka, and mouse were imported into Consensus PathDB. Dot size represents the number of DEGs represented in that function and dot color represents statistical significance measured by the *p*-value (red is a lower *p*-value and white is a higher *p*-value). **Figure S4:** Comparison of FL-incited DEG expression patterns to literature reported transcriptomics datasets. Mouse skin FL-incited DEGs were compared to DEGs modulated by Imiquimod (IMQ), a compound that is used to induce psoriasis-like skin inflammation, in mouse skin (dark blue); mouse liver FL incited DEGs were compared to DEGs modulated by Lipopolysaccharide (LPS) challenge-modulated genes in mouse liver (red); and zebrafish liver FL incited DEGs were compared to DEGs modulated by *Edwardsiella tarda* vaccination in zebrafish liver (light blue). Genes that are present in both the FL-modulated DEGs and each literature reported DEGs are plotted as a dot plot, with each color representing a particular dataset comparison, and each dot representing a shared gene between two datasets. The position of each dot is defined by X and Y values. The X value is the Log_2_(Fold Change) (Log_2_(FC)) following FL exposure, and the Y value is the Log_2_(FC) reported by a particular treatment performed in literature. Dots that fall into the X > 0, Y > 0 or X < 0, Y < 0 means FL exposure and treatment performed in the literature led to the same direction of modulation of a gene’s expression; dots that fall into the X > 0, Y < 0 or X < 0, Y > 0 means FL exposure and treatment performed in the literature led to an opposite modulation of gene expression. **Table S1:** Custom NanoString probes used to confirm the zebrafish RNA-Seq results. **Table S2:** Read depth and RNA-Seq statistics for FL-exposed and sham-treated zebrafish, medaka, and mouse samples. **Table S3A–C:** Differentially modulated genes for zebrafish (**A**), medaka (**B**), and mouse (**C**) skin samples as determined by the EdgeR with a |log_2_(FC)| ≥ 1.0 (FDR < 0.05). **Table S4A–C:** Differentially modulated genes for zebrafish (**A**), medaka (**B**), and mouse (**C**) brain samples as determined by the EdgeR with a |log_2_(FC)| ≥ 1.0 (FDR < 0.05). **Table S5A–C:** Differentially modulated genes for zebrafish (**A**), medaka (**B**), and mouse (**C**) liver samples as determined by the EdgeR with a |log_2_(FC)| ≥ 1.0 (FDR < 0.05).
