# Supplementary material for: Fluorescent Light Incites a Conserved Immune and Inflammatory Genetic Response within Vertebrate Organs (Danio rerio, Oryzias latipes and Mus musculus)
Source: Genes (Basel). 2019 Apr 3;10(4):271. doi: 10.3390/genes10040271 (PMC6523474; doi:10.3390/genes10040271)

Figure S1- Skin

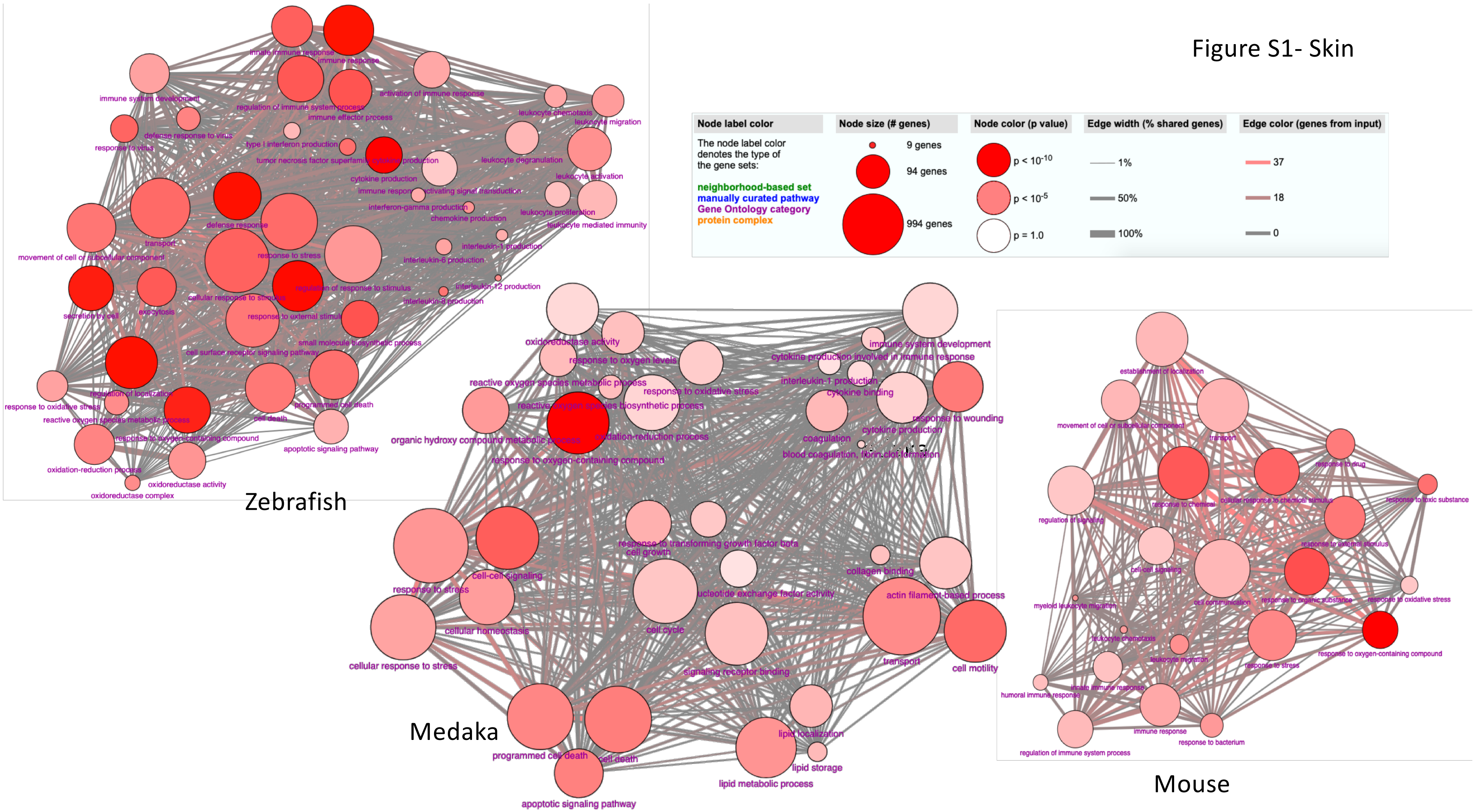

Supplement: Supplementary file 1 [file genes-10-00271-s001.zip › Genes_Supp_Mat_Sub/Supplemental Figure 1.pdf]
