# Supplementary material for: Fluorescent Light Incites a Conserved Immune and Inflammatory Genetic Response within Vertebrate Organs (Danio rerio, Oryzias latipes and Mus musculus)
Source: Genes (Basel). 2019 Apr 3;10(4):271. doi: 10.3390/genes10040271 (PMC6523474; doi:10.3390/genes10040271)
Supplement: Supplementary file 1 [file genes-10-00271-s001.zip › Genes_Supp_Mat_Sub/Table S2.docx]

Table S2: Read depth and RNA-Seq statistics for FL exposed and sham treated samples.

| Species | Organ | Filtered Reads (x10^7^) | Read Length (x10^9^) | Reads Mapped (x10^7^) | Reads Mapped (%) | Coverage |
| --- | --- | --- | --- | --- | --- | --- |
| Zebrafish FL | Skin | 6.70 | 7.3 | 5.46 | 81.5 | 120.9 |
|  |  | 7.15 | 7.8 | 5.81 | 81.2 | 129.4 |
|  | Brain | 5.10 | 6.0 | 4.03 | 79.0 | 99.6 |
|  |  | 5.14 | 6.0 | 4.05 | 78.7 | 99.5 |
|  | Liver | 5.77 | 6.9 | 4.87 | 84.4 | 113.5 |
|  |  | 4.78 | 5.6 | 40.6 | 85.0 | 93.1 |
| Zebrafish Sham | Skin | 7.87 | 8.6 | 6.47 | 82.2 | 142.7 |
|  |  | 7.50 | 8.2 | 6.14 | 81.8 | 135.7 |
|  | Brain | 5.49 | 6.4 | 4.33 | 78.7 | 106.0 |
|  |  | 5.51 | 6.4 | 4.35 | 78.9 | 106.5 |
|  | Liver | 4.72 | 5.6 | 3.98 | 84.4 | 91.9 |
|  |  | 4.89 | 5.8 | 4.15 | 84.9 | 95.3 |
| Medaka FL | Skin | 4.83 | 5.4 | 4.07 | 84.2 | 165.8 |
|  |  | 4.74 | 5.1 | 4.02 | 84.8 | 158.6 |
|  | Brain | 4.59 | 5.4 | 3.85 | 83.9 | 166.6 |
|  |  | 5.03 | 5.5 | 4.25 | 84.6 | 170.2 |
|  | Liver | 5.24 | 5.8 | 4.44 | 84.7 | 178.0 |
|  |  | 5.42 | 5.9 | 4.59 | 84.8 | 183.9 |
| Medaka Sham | Skin | 4.96 | 5.4 | 4.19 | 84.6 | 167.7 |
|  |  | 4.90 | 5.4 | 4.11 | 84.0 | 167.8 |
|  | Brain | 4.64 | 5.2 | 3.89 | 83.8 | 161.4 |
|  |  | 4.36 | 5.1 | 3.65 | 83.7 | 158.8 |
|  | Liver | 4.45 | 4.9 | 3.77 | 84.7 | 150.6 |
|  |  | 4.11 | 4.8 | 3.42 | 83.2 | 146.9 |
| Mouse FL | Skin | 5.89 | 7.9 | 5.40 | 91.7 | 131.7 |
|  |  | 5.79 | 7.8 | 5.28 | 91.2 | 129.4 |
|  | Brain | 8.39 | 12.1 | 7.52 | 89.6 | 202.5 |
|  |  | 7.92 | 11.4 | 7.13 | 90.0 | 190.1 |
|  | Liver | 8.08 | 11.5 | 6.79 | 84.0 | 192.1 |
|  |  | 8.94 | 12.9 | 7.62 | 85.3 | 215.2 |
| Mouse Sham | Skin | 5.45 | 7.4 | 4.97 | 91.1 | 123.1 |
|  |  | 7.63 | 10.4 | 6.96 | 91.2 | 173.3 |
|  | Brain | 7.65 | 11.0 | 6.87 | 89.9 | 184.3 |
|  |  | 7.69 | 11.0 | 6.92 | 90.0 | 184.2 |
|  | Liver | 8.53 | 12.3 | 7.17 | 84.1 | 205.1 |
|  |  | 8.60 | 12.4 | 7.28 | 84.6 | 207.5 |
