# Supplementary material for: Fluorescent Light Incites a Conserved Immune and Inflammatory Genetic Response within Vertebrate Organs (Danio rerio, Oryzias latipes and Mus musculus)
Source: Genes (Basel). 2019 Apr 3;10(4):271. doi: 10.3390/genes10040271 (PMC6523474; doi:10.3390/genes10040271)
Supplement: Supplementary file 1 [file genes-10-00271-s001.zip › Genes_Supp_Mat_Sub/SUPPLEMENTAL FIG_TABLE_legends.docx]

**SUPPLEMENTAL FIGURES**

**Figure S1:** DEGs from FL induced skin of zebrafish, medaka and mouse were imported into Consensus PathDB. Dot size represents the number of DEGs represented in that function and dot color represents statistical significance measured by the p-value (red is a lower p-value and white is a higher p-value).

**Figure S2:** DEGs from FL induced brain of zebrafish, medaka and mouse were imported into Consensus PathDB. Dot size represents the number of DEGs represented in that function and dot color represents statistical significance measured by the p-value (red is a lower p-value and white is a higher p-value).

**Figure S3:** DEGs from FL induced liver of zebrafish, medaka and mouse were imported into Consensus PathDB. Dot size represents the number of DEGs represented in that function and dot color represents statistical significance measured by the p-value (red is a lower p-value and white is a higher p-value).

**Figure S4:** Comparison of FL-incited DEG expression patterns to literature reported transcriptomics datasets. Mouse skin FL incited DEGs were compared to DEGs modulated by Imiquimod (IMQ), a compound that is used to induce psoriasis-like skin inflammation, in mouse skin (dark blue); mouse liver FL incited DEGs were compared to DEGs modulated by Lipopolysaccharide (LPS) challenge modulated genes in mouse liver (red); and zebrafish liver FL incited DEGs were compared to DEGs modulated by *E. tarda* vaccination in zebrafish liver (light blue). Genes that are present in both the FL-modulated DEGs and each literature reported DEGs are plotted as a dot plot, with each color representing a particular dataset comparison, and each dot representing a shared gene between two datasets. The position of each dot is defined by X and Y values. The X value is the Log_2_(FC) following FL exposure, and the Y value is the Log_2_(FC) reported by a particular treatment performed in literature. Dots that fall into the X>0, Y>0 or X<0, Y<0 means FL exposure and treatment performed in the literature led to the same direction of modulation of a gene’s expression; Dots that fall into the X>0, Y<0 or X<0, Y>0 means FL exposure and treatment performed in the literature led to an opposite modulation of gene expression.

**SUPPLEMENTAL TABLES**

**Table S1:** Custom NanoString probes used to confirm the zebrafish RNA-Seq results.

**Table S2:** Read depth and RNA-Seq statistics for FL exposed and sham treated zebrafish, medaka and mouse samples.

**Table S3A-C:** Differentially modulated genes for zebrafish (A), medaka (B) and mouse (C) skin samples as determined by the EdgeR with a |log_2_(fold change)| ≥ 1.0 (FDR < 0.05). All genes above the solid line were used for IPA analysis and genes below the solid line were either not mapped by IPA or not assigned a HUGO ID.

**Table S4A-C:** Differentially modulated genes for zebrafish (A), medaka (B) and mouse (C) brain samples as determined by the EdgeR with a |log_2_(fold change)| ≥ 1.0 (FDR < 0.05). All genes above the solid line were used for IPA analysis and genes below the solid line were either not mapped by IPA or not assigned a HUGO ID.

**Table S5A-C:** Differentially modulated genes for zebrafish (A), medaka (B) and mouse (C) liver samples as determined by the EdgeR with a |log_2_(fold change)| ≥ 1.0 (FDR < 0.05). All genes above the solid line were used for IPA analysis and genes below the solid line were either not mapped by IPA or not assigned a HUGO ID.
